# Analysis of Tetracyclines in Medicated Feed for Food Animal Production by HPLC-MS/MS

**DOI:** 10.3390/antibiotics5010001

**Published:** 2015-12-24

**Authors:** Rosa Elvira Gavilán, Carolina Nebot, Jose Manuel Miranda, Yolanda Martín-Gómez, Beatriz Vázquez-Belda, Carlos Manuel Franco, Alberto Cepeda

**Affiliations:** 1 Department of Analytical Chemistry, Nutrition and Bromatology, Faculty of Veterinary Medicine, University of Santiago de Compostela, 27002 Lugo, Spain; Rosaelvira.gavilan@rai.usc.es (R.E.G.); josemanuel.miranda@usc.es (J.M.M.); beatriz.vazquez@usc.es (B.V.-B.); carlos.franco@usc.es (C.M.F.); cepeda@usc.es (A.C.); 2 Laboratorio de Sanidad Animal, Lugar Barrio Jove de Arriba, 0 S/N, 33290 Gijón, Spain; yolanda.martingomez@asturias.org

**Keywords:** feed, antimicrobial, tetracyclines, HPLC-MS/MS, Decision 2002/657/EC

## Abstract

The use of medicated feed is a common practice in animal food production to improve animal health. Tetracyclines and β-Lactams are the groups that are most frequently added to this type of feed. The measurement of the concentration of the analytes in these types of samples is sometimes due to the matrix characteristic, and manufacturers are demanding fast, precise and reproducible methods. A rapid confirmatory method based on a simple extraction protocol using acidified methanol and followed by high performance liquid chromatography coupled to a tandem mass spectrometer for the quantification of four tetracyclines in feed is presented. Validation was performed following the guidelines of Decision 2002/657/EC. Results indicated that the four tetracyclines can be identified and quantified in a concentration range between 50 and 500 mg/kg with recoveries between 84% and 109% and RSD for precision under reproducible conditions between 12% and 16%. Satisfactory results were also obtained with interlaboratory studies and by comparing the method with an HPLC-Fluorescent method.

## 1. Introduction

Meat consumption increases each year and, consequently, so does food production of animal origin [1]. To increase production and reduce cost, animals are raised intensively in farms; big farms require greater control of animal health because illnesses can be easily transmitted from one animal to another and cause large economic losses. Therefore, the use of veterinary drugs in food production is very important for controlling and improving animal health. These substances are not only used for therapeutic treatment but also for prophylaxis.

Pharmaceuticals can be administered to animals in various forms including tablets, suspensions, emulsions, injections, implants and creams. A common practice is the administration of pharmaceuticals for prophylactic purposes through food. Intensively produced animals are often fed with concentrated feed, which are a mixture of various materials (oats, wheat, barley, rye, cottonseed, and crambe) and additives. Various classes of veterinary medicines are administered through feed as prophylactic treatment, including antibiotics (sulphonamides, tetracyclines and β-Lactams) and anti-parasitic agents (coccidiostats and ivermectins). As in humans, the pharmaceutical dose will depend on the species of animal. Given that the same maker produces feed for various species of animals, an exhaustive quality control of the concentration of the pharmaceutical in the feed should be carried out. According to Kools *et al.* (2008), the estimation of pharmaceuticals used in food production was 6.051 t of active substance for the European Union. Antibiotics was the most frequently used group (5393 t) followed by anti-parasitic agents (194 t) [2]. The study also indicated that within the antibiotics class, tetracyclines and β-lactams were the group used in the highest amounts. Most controls related to veterinary drugs in animal food production are performed on the final food (egg, milk, muscle, liver, *etc.*). Some controls are also conducted on water for animals, their food, and their faeces, but most of the analyses are for the evaluation of the presence of substances such as pesticides [3,4], nitrofurans [5] and mycotoxins [6,7,8].

The presence of substances such as antibiotics in feed samples is normally assessed by the manufacturer and generally with screening methods. Few analytical methods can be found in the literature for antimicrobials in medicated feed [9,10,11] and most of the methods reported describe analytical techniques for non-medicated feed or residues of antibacterial in food [12,13,14]. Administration of medicated feed to animal food production can only be conducted under veterinary prescription and vigilance therefore control of the correct antimicrobial concentration in the feed is vital to avoid further feed safety problems. Additionally, it should be highlighted that residue of tetracycline in feed for animal food production are not permitted at any concentration.

Based on the common use of tetracycline in food production, the low number of methods available for their analysis in feed for animal food production samples and the manufacture demand, the aim of this research work is to present an reliable and producible HPLC-MS/MS method for the analysis of tetracyclines in medicated feed samples.

## 2. Results

To optimize each tetracycline standard solutions of individual compounds at 1 mg/L in 0.1% formic acid in methanol were infused into the MS. This helped to select the precursor and product ions of each of the tetracyclines and the internal standard (IS). The cone voltage and collision energy were optimised to obtain the most intense signal for each ion. Table 1 shows precursor and product ions selected for tetracycline identification, as well as the cone voltage and collision energy employed for each transition. The transition between the precursor ion and product ion 1 and 2 was employed for confirmation of the analytes, and the transition between the precursor ion and product ion 1 was employed for quantification.

**Table 1:** Retention time, cone voltage, collision energy, precursor and product ions employed for ion identification.

	Retention Time (min)	Precursor > Product Ion	Cone Voltage (V)	Collision Energy
Tetracycline	11.71	445 > 410	30	29
		445 > 154	30	27
		445 > 226	30	27
Doxycycline	13.41	445 > 428	30	20
		445 > 201	30	27
		445 > 153	30	20
Clortetracycline	11.84	479 > 260	30	23
		479 > 286	30	23
		479 > 305	30	23
Oxytetracycline	12.61	461 > 426	30	20
		461 > 443	30	20
		461 > 408	30	20
Democlociclina	12.00	465 > 448	30	17
		465 > 288	30	17

The selection of the HPLC column and the chromatography method was based on previously described work [15]; however, the gradient was modified to achieve better resolution between the eluted peaks.

The best results were achieved with the simplest extraction protocol which employed acidified methanol; this protocol was previously described by AOAC for the extraction of one tetracycline in feed samples [16]. Once the method was selected, the validation procedure was conducted.

**Figure 1:**
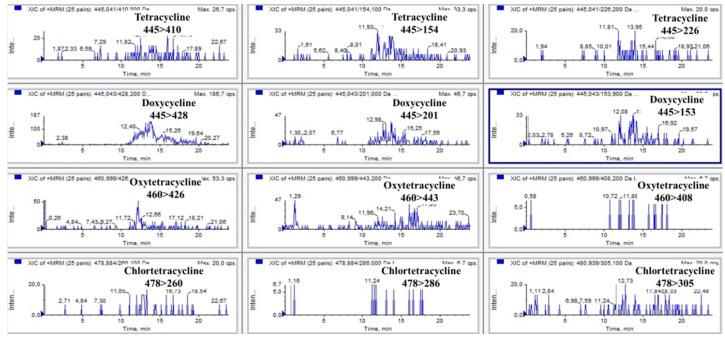
SRM chromatograms of tetracyclines in a blank sample.

**Figure 2:**
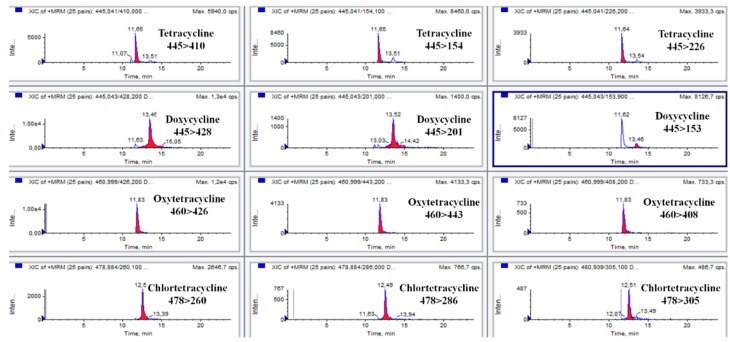
SRM chromatograms of tetracyclines in a sample fortified at 50 mg/kg.

The validation was conducted following the requirement included in the Decision 2002/657/EC [17]. A total of 20 feed samples were analysed to determine selectivity/specificity. The successful quantification of tetracyclines and the absence of interfering peaks at their retention times demonstrated the selectivity/specificity of the method. Figure 1 and Figure 2 show SRM chromatograms of tetracyclines in a blank sample and in a fortified sample at 50 mg/kg.

**Table 2:** Recoveries (%), repeatability (RSD%), within-laboratory reproducibility (RSD%) achieved with the validation of the HPLC-MSMS method and at 50 mg/kg with the HPLC-Fluorescent method.

(mg/kg)	HPLC-MS/MS				HPLC-Fluorescent			
	Accuracy	Recoveries	Repeatability	Reproducibility	Accuracy	Recoveries	Repeatability	Reproducibility
Clortetracycline								
50	103	98	8	10	98	62	2.4	7.3
100	90		7	8				
150	94		7	8				
Oxitetracycline								
50	93	95	9	14	101	60	3.7	3.9
100	102		8	8				
150	95		7	9				
Tetracycline								
50	104	98	8	13	108	50	3.4	6.7
100	104		11	13				
150	109		4	7				
Doxycycline								
50	87	92	15	16	108	58	1.9	6.9
100	94		11	12				
150	84		10	12				

Reference materials were not available; therefore, the accuracy of the method was calculated in terms of recoveries. The precision, defined as the closeness of agreement between independent test/measurement results obtained under stipulated conditions, was calculated under repeatability and reproducibility conditions, in which case it was called the repeatability or reproducibility of the method. The recoveries and the precision of the method were calculated by employing feed samples spiked with tetracyclines at 50, 100, 150 mg/kg, with six replicates for each concentration, with repeatability analysis conducted on the same day and reproducibility analysis conducted over different days. Recoveries, repeatability, and within-laboratory reproducibility achieved for each of the tetracyclines are summarised in Table 2.

Tetracycline showed the lowest recovery variation between concentrations and chlortetracycline the highest recovery variation. Although tetracycline recoveries were between 84% and 109%, this was considered an acceptable value and was within the limits set up by the Commission Decision 2002/657/EC [17]. The maximum RSD for precision under reproducible conditions accepted for concentrations of 100 mg/kg is 23 and RSD for precision under repeatable conditions should be between 11.5 and 15.6. The lowest repeatability values were achieved for chlortetracycline, with RSD below 9, and doxycycline had the highest RSD (15%). RSD for within-laboratory reproducibility were between 7% (tetracycline) and 16% (doxycycline). Therefore, it could be said that RSD for precision under repeatability and reproducibility conditions are within the limits set up by the Commission Decision 2002/57/EC.

Limit of detection and limit of quantification of the method were calculated and verified with feed samples spiked with the tetracyclines at different concentrations. This was based on a signal to noise ratio above 3 for the limit of detection (LOD), and above 10 for the limit of quantification (LOQ). The LOD and LOQ for this method should be between 1 and 10 mg/kg; however, considering the fact that the extract was diluted ten times before analysis, the LOD and LOQ should be at least ten times lower. On the other hand, the method was optimised for the extraction of tetracyclines at mg/kg levels; therefore, an LOD and LOQ at the range of µg/kg could be interesting for other types of analyses but not in this work. Considering validation results, peak shape, repeatability, reproducibility and accuracy, the LOD and LOQ of the method have been established at 5 mg/kg for all tetracyclines. To verify this, LOD feed samples spiked with tetracyclines at 1, 5, 10 and 20 mg/kg were extracted and analysed, these extract were not diluted, they were injected after filtration. The signal to noise ratio of each concentration for the individual tetracycline was investigated. A signal to noise ratio above 10 was achieved for each tetracycline at 5 mg/kg.

## 3. Discussion

### 3.1. HPLC-MS/MS Analysis

The fact that concentrations of tetracycline in medicated feed are within the range of mg/kg simplifies their extraction for HPLC-MS/MS analysis. This technique has been selected because it is defined as a confirmatory technique by the Commission Decision 2002/657/EC.

The analysis of these antibiotics in medicated feed has been previously described, employing other techniques such as synchronous spectrofluorimetry [18], and HPLC-UV [19,20]. HPLC-MS/MS methods have been also been used for tetracycline in feed, but at trace levels [21,22].

Before establishing the final extraction protocol, previously reported sample treatments for tetracycline analysis in food and feed matrices were tested. Considering that tetracyclines form chelation complexes with different cations, the use of EDTA is a common practice and has been reported for their analysis in food samples such as liver, muscle, and fish muscle [14,18,22,23]. The McIlvaine buffer, a citrate/phosphate buffer, is also commonly used for tetracycline analysis with or without EDTA [13,22,23,24,25]. These two agents were used separately, combined and with the addition of trichloroacetic acid for the extraction of tetracyclines in animal feed. After this first extraction, the supernatant was mixed with different amounts of ethyl acetate and the organic layer was evaporated to dryness. The dry extract was reconstituted in a 90:10 mobile phase A:B for analysis. However, none of the combinations tested gave satisfactory results according to data reported by the manufacturer.

Tetracyclines dissolve well in alcohol, but methanol is not commonly used for their extraction because its clean-up is difficult. A method reported by Phenomenex for honey samples which employs acidified methanol, 0.833 mL of 1 M HCl in 200 mL of methanol, followed by purification of the extract with SPE, also produced unsatisfactory results.

The best results were achieved with the simplest extraction protocol, which employed acidified methanol; this protocol was previously described by AOAC for the extraction of one tetracycline in feed samples [16]. Methanol was acidified at a higher level than conditions reported by Phenomenex and dilution of the extract into the mobile phase for analysis. It was also noted that an important factor on the extraction of tetracycline from feed samples was the shaking time for this particular method; the best recoveries were achieved for concentrations between 50 and 500 mg/kg by shaking the samples for 20 min in an orbital shaker. Shorter shaking times considerably reduced the recoveries, but higher times did not increase the recoveries.

### 3.2. Comparison between HPLC-MS/MS and HPLC-Fluorescent Detection

The results obtained with validation of the HPLC-MS/MS method were compared with those obtained with an HPLC-Fluorescent method (Table 2). The main difference between the two methods is detection of the compounds, because the extraction protocol is the same. Data on accuracy were very similar for both methods; at 50 mg/kg, the accuracy for the MS/MS method was from 87% (doxicicline) to 104% (tetracycline) and for the fluorescent method it was from 98% (chlortetracycline) to 108% (tetracycline). Precision under repeatability and reproducibility conditions achieved with the HPLC-Fluorescent method were, in general, three times lower than those obtained during validation of the HPLC-MS/MS method (Table 2). These results could be probably due to factors such as dilution; the HPLC-MS/MS method required a dilution of the extract prior to its analysis. Additionally, while the HPLC-Fluorescent technique is direct analysis, MS detection required a gas phase of the compounds which was achieved by electrospray ionisation, implying more variation between samples.

### 3.3. Interlaboratory Studies

Feed samples prepared by the manufacturer with known concentrations of individual tetracyclines were analysed by the HPLC-MS/MS method presented and by the HPLC-fluorescent method for comparison. Satisfactory results were achieved; concentrations similar to those added to the feed samples were measured with both methods (accuracy in both laboratories were between 89% and 105%). Additionally, the laboratories took part in different interlaboratory studies; accuracy was between 85% and 102% for concentrations between 20 and 100 mg/kg and values of z-score were between −1.77 and 1.5.

## 4. Material and Methods

### 4.1. Chemicals, Reagents and Stock Solutions

The chemical and chromatographic reagents used were LC or analytical grade. Tetracycline, chlortetracycline, doxycycline, oxytetracycline and demeclocycline, all with purity greater than 98%, were obtained from Sigma-Aldrich (St Louis, MO, USA). Demeclocycline was used as IS.

Disodium hydrogen phosphate dehydrate, anhydrous citric acid, ethylenediaminetetraacetic acid disodium salt (EDTA), trichloroacetic acid (TCA) and formic acid (purity >99% by analysis) were purchased from Sigma-Aldrich (St Louis, MO, USA). Organic solvents, such as methanol and ethyl acetate, were purchased from Scharlau Chemie (Barcelona, Spain). Demineralised water (resistivity 18 MU cm) was obtained in-house with a Milli-Q water system (Millipore, Bedford, MA, USA).

To prepare the mobile phase A, 400 µL of formic acid was dissolved in 9960 µL of Milli-Q water, and to prepare mobile phase B, 400 µL of formic acid was dissolved in 99,600 µL of methanol.

Individual stock solutions of tetracyclines were prepared by dissolving 20 mg of each analyte in 20 mL of methanol and stored at −20 °C for six months. These solutions were then mixed together and diluted with methanol to obtain a solution of intermediate concentration of all tetracyclines at 50 mg/L, which was stored at −20 °C for no more than one month. Each day, working standard solutions of all tetracyclines were prepared by diluting each intermediate solution in methanol to obtain a final concentration of 1 mg/L. Stock, intermediate and working solutions of the IS were also prepared and stored under the same conditions as the tetracyclines.

Extraction solution was prepared by dissolving 980 mL of methanol and 20 mL of concentrated hydrochloric acid.

### 4.2. Analysis by HPLC-MS/MS

The HPLC-MS/MS system consisted of an Alliance 2795 HPLC and Quattro Premier XE triple quadrupole mass spectrometer, both from Micromass (Manchester, UK). The software Masslynx 4.1, also from Micromass (Manchester, UK), was employed to acquire the data and control the system. The analyses of the tetracyclines were performed using a Sunfire C18 column (150 × 2.1 mm i.d., 5.0 mm particle size) from Waters (Milford, MA, USA). Separation of the analytes was achieved by injecting 25 µL of extract and by applying a mixture of component A (0.04% formic acid in water) and B (0.04% formic acid in methanol) on a gradient mode as follows: 0–8 min 0% B, 8–9 min 45% B, 9–14 min 61% B, 14–15 min 0% B, and 15–18 min 0% B. The oven temperature was set at 35 °C, and a flow rate of 0.25 mL·min^−1^ was used during the whole run.

Separated tetracyclines were directed into the electrospray source of the MS, which was operated in the positive-ion mode and under the following conditions: capillary voltage 3 kV, source temperature 120 °C, desolvation temperature 350 °C, cone gas flow 49 L/h, and desolvation gas flow 650 L/h. The analytes were identified by their retention times (Rt) and by 2 or more selected reaction monitoring (SRM) transitions.

### 4.3. Sample Extraction for HPLC-MS/MS Analysis

Sample preparation procedure was based on a previous work (AOAC 2009). Approximately 200 g of feed was ground and homogenised in a Moulinex grinder and 2 g of the powder was transferred into a 50 mL disposable plastic centrifuge tube. To extract the tetracyclines form the feed, 20 mL of extraction solution was added, and the tube was closed and shaken for 20 min at 200 rpm on a New Brunswick Scientific model G25 shaker (New Jersey, NJ, USA). The mixture was centrifuged at 2500 rpm on a model 5415D centrifuge from Eppendorf (Hamburg, Germany) and 500 µL of the extract was then filtered through an Ultrafree-MC centrifugal filter from Millipore. The filtered extract was diluted ten times with mobile phase mixture 90A:10B and transferred into an amber HPLC vial that contained a 300 mL micro-insert and was kept at −18 °C until sample analysis by HPLC-MS/MS, which took place within 24 h.

For quantification, blank feed samples (feed without tetracyclines) were fortified by spiking them with different aliquots of a standard mixture of tetracyclines to obtain the following final concentrations: 50, 100, 150, 200 and 250 mg/kg. After fortification, and prior to extraction, samples were shaken on an orbital shaker at 200 rpm for 10 min. In addition to these, two samples containing only the reagents (without feed) were also processed. One was spiked with tetracycline at 100 mg/kg (fortified reagents), and the other was not spiked (reagent blank), it simply contained reagents.

For method the method development feed samples spiked with tetracycline at various concentrations were employed (from 1 to 300 mg/kg). These samples were extracted employing the protocol described above and the linearity of the whole protocol was investigated. Taking into account the fact that concentration of tetracyclines in medicated feed are 100 mg/kg, validation was conducted at 150 mg/kg.

### 4.4. Validation of the HPLC-MS/MS Method

Commission Decision 2002/657/EC establishes the criteria and results interpretation for methods for analysis of samples of food and food-producing animals. Even though the method has been used for the analysis of medicated feed samples, the criteria included in Commission Decision 2002/657/EC were followed for the identification and quantification of chlortetracycline, doxycycline, oxytetracycline and tetracycline in feed samples (EU 2002). However, as maximum residual levels for these substances are not applicable detection limits (CCβ), the decision limit (CCα) of the method was not investigated. On the other hand, trueness/recovery, precision, specificity and applicability/ruggedness/stability were investigated in two different laboratories dedicated to the control of residue of veterinary drugs in food samples.

For validation of the method three batches of matrix-matched samples were employed. Each batch, analysed on a different day, consisted of 21 samples fortified with tetracyclines at concentrations of 0, 50, 100, 150, 200 and 500 mg/kg. Six replicates were employed for levels of 50, 100, and 150 mg/kg, and only one sample was employed for levels of 0, 20 and 50 mg/kg.

For quantification, blank feed samples (feed without tetracyclines) were fortified by spiking them with different aliquots of a standard solution mixture of tetracyclines to obtain the following final concentrations: 50, 100, 150, 200 and 500 mg/kg. After fortification, and prior to extraction, samples were shaken on an orbital shaker at 200 rpm from for 10 min. In addition to these, two samples containing only the reagents (no feed) were also processed. One of these samples was spiked with tetracycline at 100 mg/kg (fortified reagents), and the other was not spiked (reagent blank) and simply contained reagents.

### 4.5. HPLC-Fluorescent Detection

This method was set up in another laboratory for comparison of the results obtained with the HPLC-MS/MS method. The HPLC-Fluerescent system consisted of an Alliance 2695 HPLC and Fluoresecen detected model 2475 both from Waters. A volume of 10 µL of extract was injected into a LiChroCART Purospher^®^ STAR RP-8 (4.6 × 150 mm, 5 μm). The mobile phase consisted of methanol (mobile phase A) and calcium chloride and EDTA buffer at pH 6.5 (mobile phase B) with a flow rate of 0.6 mL/min. The gradient program was as follows: 0 min 30% A, 8 min 55% A, 11 min 60% A, 12 min 30% A, and 17 min 30% A. The detector was operated at an excitation wavelength of 390 nm and an emission wavelength of 512 nm.

The extraction protocol employed for fluorescent detection was similar to that described above. However, in this case, extraction was performed with 20 mL of acidified methanol; after shaking them for 20 min, the extract was filtered and transferred to a 25 mL volumetric flask. The volume was made up to 25 mL with acidified methanol. As mentioned in the sample preparation section, the extract was filtered prior to HPLC-fluorescent detection.

### 4.6. Interlaboratory Studies

For interlaboratory studies, samples were obtained from different sources including feed manufacture and from the Association of American Feed Control Officials (AAFCO) and INTER2000. Medicated feed samples supplied from manufacture contained 70 mg/kg of chlortetracycline, 50 mg/kg chlortetracycline, oxitetracycline 50 mg/kg and 200 mg/kg of tetracycline. Those samples obtained from the Association of American Feed Control Officials (AAFCO) and INTER2000 contained chlortetracycline 80 mg/kg, doxycycline 29 mg/kg, oxytetracycline 400 mg/kg, chlortetracycline 60 mg/kg, chlortetracycline 55 mg/kg and oxytetracycline 28 mg/kg.

## 5. Conclusions

The use of veterinary medicine in animal food production is a common practice; pharmaceuticals are used for therapeutic and prophylactic proposals. A simple way to give antimicrobials to the animals is through food; however, few methods exist for the accurate analysis of these legal substances in feed samples. The present work describes a HPLC-MS/MS method for the simultaneous analysis of four tetracyclines in feed samples; the results were compared with an HPLC-Fluorescent method which employed the same extraction protocol. The method produced satisfactory results for concentrations of tetracyclines between 50 and 500 mg/kg.
